# Sea lice, *Lepeophtheirus salmonis* (Krøyer 1837), infected Atlantic salmon (*Salmo salar* L.) are more susceptible to infectious salmon anemia virus

**DOI:** 10.1371/journal.pone.0209178

**Published:** 2019-01-16

**Authors:** Sarah E. Barker, Ian R. Bricknell, Julia Covello, Sarah Purcell, Mark D. Fast, William Wolters, Deborah A. Bouchard

**Affiliations:** 1 Aquaculture Research Institute, University of Maine, Hitchner Hall, Orono, Maine, United States of America; 2 School of Marine Sciences, The University of Maine, Hitchner Hall, Orono, Maine, United States of America; 3 Hoplite Lab, Department of Pathology and Microbiology, Atlantic Veterinary College, University of Prince Edward Island, Charlottetown, PEI, Canada; 4 USDA ARS National Cold Water Marine Aquaculture Center, Franklin, Maine, United States of America; Texas A&M University, UNITED STATES

## Abstract

The role of parasitic sea lice (Siphonostomatoida; Caligidae), especially *Lepeophtheirus salmonis*, in the epidemiology of Infectious Salmon Anemia Virus (ISAv) has long been suspected. The epidemiological studies conducted during the 1998 major Infectious Salmon Anaemia (ISA) outbreak in Scotland demonstrated a strong correlation between sea lice presence and ISAv positive sites or subsequent clinical outbreaks of ISA. The question posed from this observation was “do sea lice infestations on Atlantic salmon make them more susceptible to viral infections?”

This study investigated the role that sea lice infestations have on the severity of ISAv infections and disease mortality in experimental populations of farmed Atlantic salmon (Salmo salar). A series of experiments was carried out that investigated the potential of sea lice to modify the outcome of an ISAv infection. Experimental populations of Atlantic salmon were established that had: no lice and no ISAv, a single infection with either ISAv or lice and a co-infection with lice then ISAV. The results were quite clear, the process of infestation by the parasite prior to ISAv exposure significantly increased the mortality and death rates of Atlantic salmon, when compared to uninfected controls and ISAv infected groups only. This was consistent over two source strains of Atlantic salmon (Pennobscot and Saint John River), but the severity and timing was altered. Immunological responses were also consistent in that pro-inflammatory genes were induced in lice only and co-infected fish, whereas the anti-viral response, Mx, MH class I β, Galectin 9 and TRIM 16, 25 genes were down-regulated by lice infection prior to and shortly after co-infection with ISAv. It is concluded that the sea lice settlement on Atlantic salmon and the parasite’s subsequent manipulation of the host’s immune system, which increases parasite settlement success, also increased susceptibility to ISAv.

## Introduction

The salmon louse, *Lepeophtheirus salmonis* (Krøyer 1837), and infectious salmon anemia virus (ISAv) are the two most significant pathogens of the Atlantic salmon (*Salmo salar*, L) aquaculture industry. *L*. *salmonis* has major economic consequences for the industry and estimates vary from approximately US$500 million to roughly $1billion in losses worldwide [1–5].

Sea lice have a complex life cycle consisting of three planktonic stages, nauplius I and nauplius II followed by the host seeking copepodid stage. Following attachment of the copepodid stage the parasite molts into the attached chalimus I and II stages, where it feeds on the epidermis of the fish. This is followed by a molt into the mobile pre-adult I and subsequently the pre adult II stage, before finally molting to the sexually mature adult stage. The life cycle is temperature dependent and can take as little as 28 days at 14°C to several months at colder temperatures.

The planktonic stages cause little damage to the host, although it has been suggested that copepodids would take a small meal prior to attaching to the host [6]. The two sessile chalimus stages only cause minor damage to the host and the damage is limited to the epidermis. Damage is caused by the extrusion and attachment of the frontal filament and local tissue erosion as the parasite feeds on the associated epithelium. More importantly, there is considerable evidence that the attached chalimus stages will down regulate the immune response of the host, which may increase the susceptibility to other pathogens and secondary infections, especially at the site of attachment [7, 8].

The pre-adult and adult stages are motile and can move between hosts quite readily. Pre-adult lice have been caught in sentinel cages of Atlantic salmon in Norway, Ireland, Canada and Scotland [9–12]. Work by Pino-Marambio et al and Mordue and Birkett [13, 14] has shown that adults and pre-adults will re-assort themselves on fish depending on the availability of potential mates with males being far more motile than females in this regard. Some work has been done looking at sea lice as vectors of disease including infectious haematopoetic necrosis virus (IHNv),[8], infectious salmon anemia virus (ISAv, [15]) and furunculosis [16]. These studies have concentrated on detecting the presence of the pathogen on or in the adult sea louse rather than a full transmission study. [15,16] The major pathological effects result from the mobile stages, which can consume large amounts of epithelial tissue forming large lesions through which secondary infection can gain access. It is feasible that if the motile stages feed on a fish that is already infected with a pathogen then it acts as a vector or fomite for that pathogen if the lice leave the infected host and settle on a naïve fish.

The ability to transmit disease between individuals may be enhanced by *L*. *salmonis’s* potential to modulate the immune system of the host. Recently there have been numerous studies that show that the adult *L salmonis* down regulates inflammatory signals and the cell mediated immune system of the host [17–22]. This is considered to be advantageous to the chalimus and mobile stages, preventing the mounting of a complement associated T_h_1, cell mediated response to the parasite. The pro-inflammatory response is the major mechanism used by *L*. *salmonis* resistant coho salmon to prevent successful louse attachment [17, 23].

Infectious salmon anemia virus is an orthomyxovirus and the etiological agent of infectious salmon anemia (ISA, [24–27]). ISA is a World Organization for Animal Health (OIE)-listed disease that has been an economic problem for the salmon farming industry in Chile, Canada, Norway, Scotland, Faroe Isles and U.S.A, [26, 28–30]. The clinical signs may include anaemia, yellow or blood-tinged ascites, a haemorrhagic liver necrosis, exophthalmia, lethargy and increased mortality [31, 32] Cumulative mortality due to ISAv infection can be highly variable from insignificant to over 90% of the fish population dependent upon the strain of ISAv, genetic background of the fish and other stressors. There is some epidemiological evidence that ISA outbreaks are associated with sea lice infestation [33, 34].

Due to the economic consequence of these two pathogens, this study investigated if *L*. *salmonis* infestations of Atlantic salmon: 1) increased susceptibility of salmon to ISAv infection, and 2) affected the systemic immune response to subsequent ISAv infection. In order to answer these questions salmon were exposed to: 1) no lice, no ISAv, 2) no lice plus ISAv and 3) lice plus ISAv 4) Lice without ISAv, and subsequent survival, ISAv prevalence and immune gene expression were recorded.

## Methods

### Ethics statement

The research presented was assessed by the University of Maine’s Animal Care and Use Committee (IACUC). All experimental design and protocols were reviewed and approved prior to any research being conducted. The University of Maine is registered as a research facility in accordance with the U.S. Department of Agriculture Animal Welfare Act and the Public Health Service Policy on the Humane Care and Use of Laboratory Animals. The University of Maine holds the Office of Laboratory Animal Welfare (OLAW) of the National Institutes of Health assurance for vertebrate animals used in research, teaching and outreach. In accordance with the Public Health Service Policy on the Humane Care and Use of Laboratory Animals, the University of Maine works with the OLAW of the National Institutes of Health to assure the welfare of animals used in research and teaching Animal Welfare, Assurance #: A3754-01. Laboratory infestations and infections of Atlantic salmon with *L*. *salmonis* and ISAv were carried out under the approved University of Maine IACUC Protocol A2012-11-06.

### Fish husbandry

Atlantic salmon (ATS) were reared from eggs at the National Cold Water Marine Aquaculture Center, Franklin, ME, and were considered specific pathogen-free based on the facility’s long history of fish health inspection screening. The ATS were Passive Integrated Transponder (PIT)-tagged intraperitoneally with 7mm PIT tags (NMT Inc) as parr in order to differentiate family status and transferred to the facility’s saltwater systems as smolts. These saltwater acclimated ATS were used to perform trials in artificial seawater at the University of Maine. Penobscot River versus Saint John River derived families were further differentiated by elastomere tagging (NMT Inc., WA, U.S.A) in order to facilitate sampling.

### Sea lice culture

Gravid, adult female *L*. *salmonis* were collected from commercial farm sites in Maine, courtesy of Cooke Aquaculture. Lice were transported in aerated natural seawater to the Aquaculture Research Institute, University of Maine sea lice hatchery in collected seawater. Egg strings were removed from the adult lice and placed in hatching chambers in a natural seawater recirculation system at 13 ± 1 °C and 33 ± 1 ppt salinity. Once larval lice had molted into the infective copepodid stage, they were collected using 64 μm mesh hatching chambers into a glass container and counted ready for bath infestation of Atlantic salmon. Only copepodids less than 4 days old were utilized for infestations.

### ISAv and ASK II cell maintenance

Atlantic salmon kidney II (ASK II) cells (Micro Technologies Inc. Richmond, ME) were maintained in Leibovitz-15 (L-15) cell culture medium (Invitrogen, MD) with 10% FBS (Gibco, Fisher Scientific, Waltham, MA) and 4 μl 2-mercapthoethanol (aids in maintaining a reducing environment, Fisher Scientific).

The Charlie Cove Back Bay (CCBB) ISAv strain (Micro Technologies Inc. Richmond, ME) was used throughout the study. This strain was isolated from infected Atlantic salmon in Charlie Cove Back Bay, New Brunswick, Canada in 1997. ISAv isolates were propagated in chinook salmon embryo (CHSE-214) cells (University of Maine cell stock collection) grown at 15 C in MEM containing 5% FBS. When cells demonstrated 75% cytopathic effect (CPE), cells and supernatant were collected and centrifuged at 1000xg for 10 min to remove cells and debris. The supernatant containing ISAv was filtered through a 0.45μm filter (EMD Millipore, MA, USA), aliquoted into 5.0 ml aliquots and stored at −80 C until required.

The ISAv inoculum titer for intra-peritoneal injection of cohabitating-fish was determined by the TCID_50_ endpoint assay in ASK cells grown at 15 C in L-15 media with 5% FBS. Briefly, the virus inoculum was diluted 1:10 in L-15 media containing gentamicin (50 μg.mL^−1^, L-15-G) and filtered through 0.45μm filters. Serial 10-fold dilutions to 10^−9^ in L-15-G media were prepared. Each L-15-G virus dilution was added in 100-μL volumes to nine wells of a 96-well plate seeded with ASK II cells. Plates were sealed with a mylar sheet and centrifuged for 30 min at 15 C at 500xg to enhance adsorption [35] Plates were subsequently incubated at 15°C. All plates were observed daily for visible CPE for 28 days. The TCID_50_ was calculated using the method of Reed and Muench [36].

### Fish husbandry

**Fig 1:**
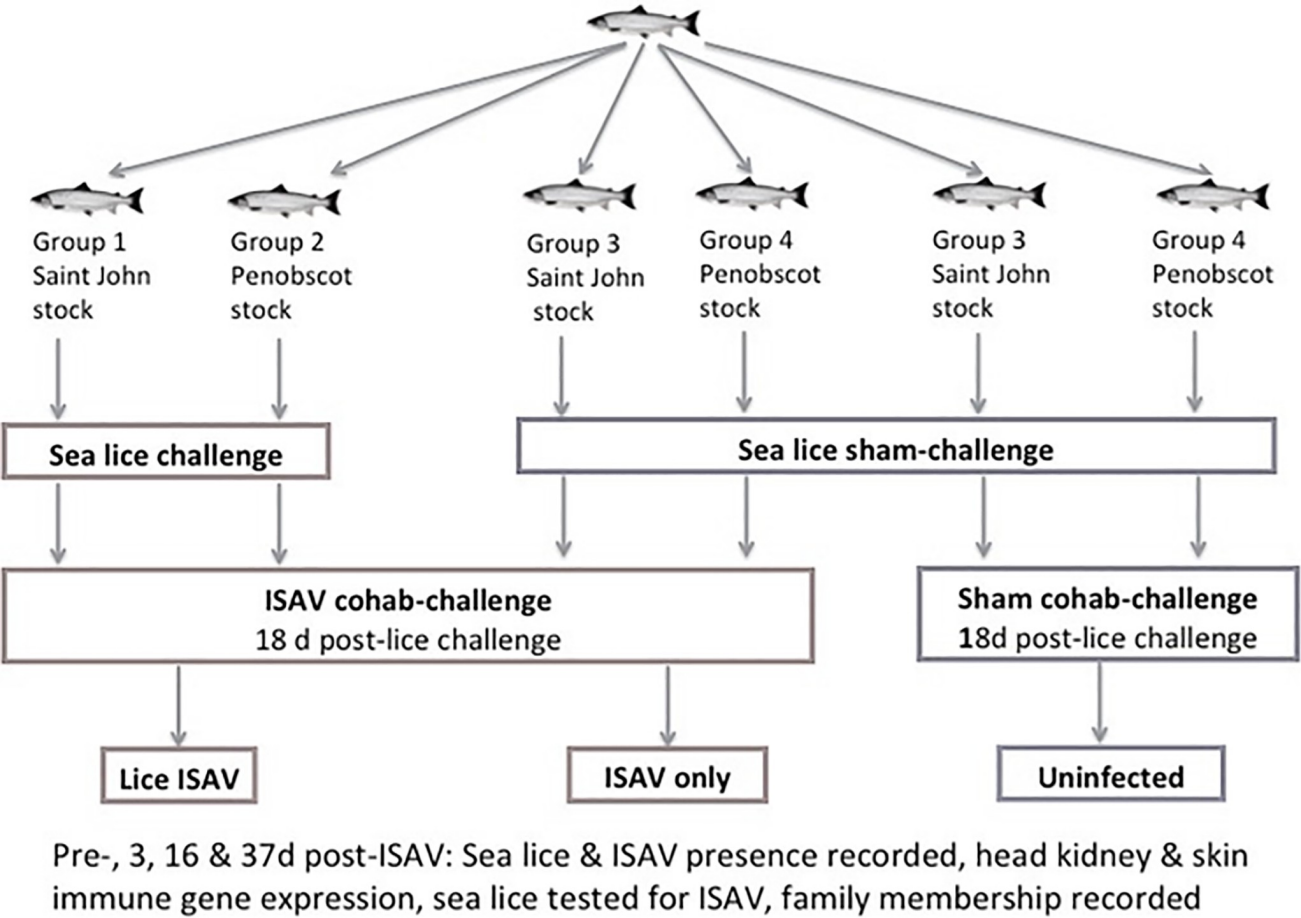
Trial outline. Three groups of Atlantic salmon from Penobscot river strain and St John river strain (six replicate tanks per treatment): sea lice infestation plus ISAv, sea lice infestations no ISAv, and no sea lice infestation no ISAv controls. Fish were bath infested with *L*. *salmonis* followed by an ISAv cohabitation infection 18 days post-lice exposure. Twenty-four fish were euthanized per treatment group (n = 12 Penobscot–derived and 12 St John–derived fish, each made up of three distinct families) at days -1, 3, 16, and 37 d.p.e., and the following parameters sampled number of lice per fish, head kidney for immune gene expression and ISAv QRT-PCR kidney, spleen and heart for cell culture. Forty-five Atlantic salmon per treatment group were also monitored until no mortalities had occurred in any group for 7 days. At 51 d.p.e. all survivor fish were sampled as detailed below.

Fish were divided into 3 treatment groups: 1) a control group, 2) an ISAv only infected group and 3) a lice + ISAv infected group (Fig 1). Four hundred and twenty-three Atlantic salmon from two strains, Penobscot- and Saint John river-derived, were randomly assigned to 18 tanks (6 replicate tanks per treatment) of an artificial saltwater recirculation system with UV sterilization. The fish were acclimatized for 3 weeks prior to commencing the trial. Each salmon strain population was made up of 3 pedigree families i.e. 6 pedigreed families in total. Water parameters were as follows: 12 ± 1 °C, 10 ± 2 ppm dissolved oxygen, 34±1 ppt salinity, room lighting was fixed at 12:12 daylight/ darkness. To prevent cross contamination at water post filtration and reoxygenation, recirculating water was subjected to UV disinfection (Emperor Aquatics 80 watt Smart UV) before the water was returned to each individual experimental tank.

### Infestation with sea lice

Fish were bath infested with *L*. *salmonis* copepodids by stopping the water flow and reducing the water to 1/3^rd^ the original tank volume modified from Bron [37]. Copepodids were added at a ratio of 150 per fish. The water flow was re-instated after 2 hours. Water temperature and dissolved oxygen were monitored continuously during the infestation procedure. The treatment groups, which did not receive a lice infection, underwent the same procedure without copepodids in order to expose the fish to the same handling process. Tank effluent flowed through 100 μm mesh bags, a bead filter and UV sterilization prior to recirculation through tanks in order to prevent unattached lice entering control tanks.

### Infection with ISAv

Once lice had begun to molt to the motile pre-adult stages, 18 days post infestation (dpi), infested, sham lice exposed, ISAv only and lice + ISAv fish groups were exposed to ISAv via a cohabitation challenge. Ten percent of the tank population were selected randomly (by use of random number tables) and were anaesthetized with 80 mg.L^-1^ MS-222, intra-peritoneal (i.p) injected with 1 x 10^4.4^ TCID_50_ ISAv per fish diluted in sterile phosphate buffered saline (PBS, 100 μl volume) and the adipose fin was clipped for identification purposes. The control group underwent the same procedure except that 10% of the population were i.p. injected with 100 μl sterile PBS only. These i.p. injected fish (ISAv^+/^ISAv^-^) were termed ‘shedder fish’ for the remainder of the study.

### Experimental design, challenge with *L*. *salmonis* followed by ISAv and sampling procedure

Fish were sampled prior to any infections occurring i.e. basal (n = 24), 17 days post infestation (dpi) to sea lice and minus 1 dpi to ISAv cohab (n = 24/group), 21 dpi to sea lice and 3 dpi to ISAv cohab (n = 24/group), 34 dpi to sea lice and 16 dpi to ISAv cohab (n = 24/group) and 55 dpi to sea lice and 37 dpi to ISAv cohab (n = 24/group, Fig 1). Survival was also monitored in duplicate tanks per treatment without sampling occurring in these tanks.

### Established humane endpoint post ISAv challenge

The use of a humane endpoint was used when necessary during the experiment. The ISAv challenge resulted in disease and mortality of fish. However, fish were monitored a minimum of three times daily post-challenge. This allowed for humane euthanasia as close in time as possible to when individuals clearly displayed signs of irreversible distress. Any moribund fish were euthanized promptly, as well as all fish exhibiting signs of distress such as respiratory gasping, listlessness, and loss of equilibrium that were determined to have reached the end stages of the disease.

### Sample collection and processing

At each sampling point fish were euthanized with 400 mg.L^-1^MS-222 (Sigma, St. Louis, MO) in individual 40 L containers. The length, weight and stock were recorded for each fish. Kidney, heart and spleen tissues were placed into a sterile Steward Stomacher Lab Blenders Bags (Fisher Thermo Scientific). The tissues were weighed, diluted 1/5 (w/v) in sterile PBS(Sigma) and homogenized. These samples were held on ice during all processing. Anterior kidney samples were excised and placed individually in RNA Later (Ambion Inc., Austin, Texas) for 48 hours at 4°C prior to removal of RNA Later and storage at -80°C until transport on dry ice to the Atlantic Veterinary College (AVC), Charlottetown, PE, Canada for gene expression analysis. Sea lice numbers on each fish were also counted and categorized as sessile (chalimus I or II), motile (pre-adult and adult) or gravid (adult female with eggs strings).

### RNA extraction and reverse transcription

Total RNA was extracted from anterior kidney samples using Trizol Reagent [38–40], 1993 according to the manufacturers’ instructions. Approximately 50 mg of tissue was added to 1.5 ml of Tri Reagent [41] and mechanically macerated with a homogenizer (VWR, Mississauga, ON). After a 5 min incubation at room temperature (RT, ~22 °C), 300 μl of chloroform (BDH, West Chester, PA) was added to each sample. Tubes were capped and shaken vigorously for 15 s. Next was a 3 min incubation at RT, the samples were centrifuged at 10,000xg for 15 min at 4°C. The aqueous phase was transferred to a new 1.5 ml tube. An equal volume of molecular grade isopropyl alcohol (Sigma) was added and the tubes were mixed by inverting 3x and then incubated for 10 min at RT. The RNA was pelleted by centrifuging at 10,000xg for 10 min at 4°C. The pellets were subsequently washed with 750 μl of ice-cold 75% (v/v) molecular grade ethanol (BDH), and centrifuged at 7,500xg for 5 min at 4°C. RNA pellets were air dried for approximately 5 min. The pellets were re-suspended in 100–200 μl of nuclease-free water before quantification on a NanoDrop-2000 spectrophotometer (Thermo Scientific, Wilmington, DE). Samples were stored at -80°C until DNase treatment.

A total of 5 μg of isolated RNA was DNase treated using a TURBO DNase-free kit (Ambion, Foster City, CA), following the manufacturers’ instructions. A random subset of RNA from each tissue was analyzed by Experion (BioRad, Hercules, CA) and all RNA Integrity Numbers (RINs) were >6.5. cDNA synthesis was performed on 1 μg of DNase treated total RNA using a Reverse Transcription System (Promega, Madison, WI) and random hexamers, according to the manufacturers’ instructions. cDNA was stored at -20°C until use in qPCR.

### Verification of ISAv infection by RT-qPCR

Primer pairs and Taqman MGB probes targeting ISAv segment 8 from Snow et al. (2006) were used for ISAv detection (Table 1). Each qPCR was run in a 96-well plate (Eppendorf, Mississauga, ON) under standard conditions in 20 μl reactions containing 1x TaqMan Gene Expression Assay (Applied Biosystems, Foster City, CA), 1x TaqMan Gene Expression Master Mix (Applied Biosystems, Foster City, CA), and 2 μl of cDNA (diluted 1:10 in nuclease-free water). A negative template control of nuclease-free water was used in the series of reactions to screen for possible cross-contamination between samples. In addition, a positive control made from identified ISAv-injected samples was run to verify detection and continuity between plates. Each sample was run in duplicate, and if the Ct values between the replicates were >0.5 cycles apart the sample was re-run on a separate plate. Assays were carried out in an Eppendorf Mastercycler ep realplex^2^ under the following conditions: initial incubation at 50°C for 2 min, then 95°C for 10 min, followed by 45 amplification cycles of denaturation at 95°C for 15s and annealing/extension at 60°C for 1 min.

**Table 1:** Real-time PCR primers and Taqman MGB probe sequences for ISAv-Segment 8 (Snow *et al*. 2006).

Upstream primer (5'-3')	Downstream Primer (5'-3')	Taqman Probe (5'-3')	Amplicon size (bp)
CTACACAGCAGGATGCAGATGT	CAGGATGCCGGAAGTCGAT	FAM-CATCGTCGCTGCAGTTC-MGB	104

### Verification of ISAv infection by ASK II cell culture

Viable ISAv was isolated by cell culture techniques with an enhanced virus adsorption technique [35]. Kidney, spleen and heart tissue homogenates were further diluted 1/10 (v/v) in L-15-G media and filtered through 0.45μm filters. Each sample was added in 100μL volumes to two wells of a 24-well plate containing ASK II cells with culture media removed from the wells. Plates were subsequently centrifuged for 30 min at 15 C at 500xg for enhanced virus adsorption, prior to the addition of 1 ml L-15 supplemented with 5% (v/v) FBS. The plates were sealed with a Mylar sheet and incubated at 15 C. All plates were monitored daily for visible CPE for 28 days. All samples with observed ISAv CPE were confirmed by RT-PCR detection of ISAv segment 8. All samples processed from mortalities and surviving fish regardless of whether CPE was observed or not, as well as 1/3^rd^ of all time point samples that were negative for CPE, were also tested by RT-PCR for confirmation purposes (Table 2).

**Table 2:** Mean percentage of Atlantic salmon positive for viable ISAv by cell culture and non-viable ISAv by QRT-PCR detection of ISAv segment 8 RNA pre- and 3, 16, 37 adm 51 days post–ISAv cohab infection. Fish sampled at 51 d.p.e. to ISAv cohab fish were survivors of the infection.

Days post-ISAv challenge	Treatment group					
	No lice No ISAv		No lice infected with ISAv		Lice plus ISAv	
	% positive for Viable ISAv	% positive for Non-viable ISAv	% positive for Viable ISAv	% positive for Non-viable ISAv	% positive for Viable ISAv	% positive for Non-viable ISAv
-1	0	0	ns	ns	0	0
3	0	0	0	0	0	0
16	0	0	0	0	35	59
37	0	9	32	42	100	100
51	0	6	61	96	85	100

Cell culture wells were scraped to dislodge the monolayer of ASK cells and the well contents aseptically collected prior to centrifugation at 20,000xg for 15 min at 4 °C. Total RNA was extracted from the pellet and approximately 250 μl cell culture supernatant using Trizol LS Reagent (Invitrogen) according to the manufacturer's instructions with the addition of 0.02 ng.μl^-1^ linear acrylamide (Ambion) to the aqueous phase prior to the addition of 500 μl molecular grade isopropanol (Fisher). Total RNA was quantified on a NanoDrop-1000 (Fisher Scientific) spectrophotometer prior to storage at -80°C.

The cDNA synthesis was performed on a total of 10 μl of isolated RNA using a High Capacity cDNA Reverse Transcription kit with RNase inhibitor (Applied Biosystems) and random hexamers, according to the manufacturers’ instructions. cDNA was stored at -20°C until PCR testing.

PCR detection of ISAv segment 8 [25] was performed using the primers outlined in Table 3. PCR was performed under standard conditions in 50μl reactions containing 5μl cDNA, 1x Go Taq flexi DNA polymerase master mix (Promega), 0.2mM each dNTPs (Promega), 0.5 μM forward and reverse primer (IDT), 1.25 U Go Taq DNA polymerase (Promega) and 1.5mM MgCl_2_ (Promega). A negative no-template control of nuclease-free water (IDT) was used in order to screen for possible cross-contamination between samples. In addition, a positive control of cDNA made from identified ISAV-injected fish cell culture samples was run to verify detection. PCR was carried out in a BioRad DNA Engine thermocycler under the following conditions: initial denaturation at 95°C for 10 min, followed by 35 amplification cycles of denaturation at 95°C for 60s, annealing at 55°C for 60 s and extension at 72 ^o^C for 60s, followed by a final elongation step at 72°C for 7 min. PCR products were resolved on a 2% (w/v) agarose gel containing 0.5 μg.ml^-1^ ethidium bromide (Fisher) with a 100bp ladder (NEB) and imaged.

**Table 3:** PCR primer sequences for ISAv-Segment 8 (Mjaaland et al., 1997).

Upstream primer (5'-3')	Downstream Primer (5'-3')	Amplicon size (bp)
GGCTATCTACCATGAACG	GCCAAGTGTAAGTAGCACTCC	157

### Immune gene expression by RT-qPCR

Primer sets for the 4 reference genes (EF-1Ab, RSP20, 18S and elF) and 7 genes of interest (Interleukin (IL)-1β, matrix metalloproteinase (MMP) 9, Mx1, major histocompatibility complex class I–beta (MCH1- β), Galectin 9, tripartite motif (TRIM) 16 and TRIM 25) were obtained from previous literature (Table 4). Each qPCR was run in a 96-well plate (Eppendorf, Mississauga, ON) combining 0.5 μl of forward primer (10 μM), 0.5 μl (10 μM) of reverse primer, 5μl of 2x GoTaq qPCR Master Mix (Promega), 3μl of nuclease-free water (Lonza, Rockland, ME) and 2 μl of cDNA (diluted 1:10 in nuclease-free water). qPCRs were performed using a CFX Connect Real-Time System (BioRad, Hercules, CA) under the following conditions: initial denaturation of 5 min at 95°C, followed by 40 amplification cycles of 95°C for 15s, annealing for 20s and extension for 30s at 72°C. Annealing temperatures varied depending on the primers used. 18 S, elF, MMP 9 and TRIM 16 were annealed at 61.6°C, RPS 20, Mx1, MHC1-b, Galectin 9 and TRIM 25 were all annealed at 64°C, and EF-1Ab and IL-1β were annealed at 65°C. At the end of each qPCR, melt curve analysis was performed to ensure that only one product was amplified.

**Table 4:** Oligonucleotide primer sets used to amplify Atlantic salmon (*Salmo salar*) reference genes (EF-1Ab, RPS 20, 18S and elF) and target genes (IL-1β, MMP 9, Mx1, MHC1-b, Galectin 9, TRIM 16 and TRIM 25) for quantitative real-time PCR (qPCR) expression studies.

Gene	Primer	Sequence (5' → 3')	Product Size	Source
EF-1A	EF1Ab F	TGCCCCTCCAGGATGTCTAC	59 bp	Olsvik *et al*, 2005
	EF1Ab R	CACGGCCCACAGGTACTG		
RPS 20	RPS20 F	GCAGACCTTATCCGTGGAGCTA	85 bp	Olsvik *et al*, 2005
	RPS20 R	TGGTGATGCGCAGAGTCTTG		
18 S	18S F	CCCCGTAATTGGAATGAGTACACTTT	98 bp	Olsvik *et al*, 2005
	18S R	ACGCTATTGGAGCTGGAATTACC		
eIF 3 subunit 6	elF F	GTCGCCGTACCAGCAGGTGATT	92 bp	Skugor *et al*, 2008
	elF R	CGTGGGCCATCTTCTTCTCGA		
IL-1β	IL-1β F	ATGCGTCACATTGCCAAC	90 bp	Fast *et al*, 2006
	IL-1β R	GGTCCTTGTCCTTGAACTCG		
MMP 9	MMP9 F	AGTCTACGGTAGCAGCAATGAAGGC	72 bp	Skugor *et al*, 2008
	MMP9 R	CGTCAAAGGTCTGGTAGGAGCGTAT		
Mx1	Mx1 F	TGCAGCTGGGAAGCAAACT	71 bp	Workenhe *et al*, 2009
	MX1 R	CAACGTTTGGCTGATCAGATTC		
MHC1-b	MHC1-b F	ACCTGAAGAGAGCGACATGGA	68 bp	Workenhe *et al*, 2009
	MHC1-b R	CCCTTCCCACTTCATTTTGGA		
Galectin 9	Gal 9 F	TCGCTGATTGTGAATGGTGCTCAC	82 bp	Jørgensen *et al*, 2008
	Gal 9 R	CAGGGTTGGAGAAGGCAATGGATT		
TRIM 16	TRIM 16 F	CTGTGTTTGGGTCCAGTGTG	114 bp	Workenhe *et al*, 2009
	TRIM 16 R	GGACCAAGATCTCCCCTACAG		
TRIM 25	TRIM 25 F	ATAGGACCCTGCCTTCACCT	115 bp	Workenhe *et al*, 2009
	TRIM 25R	CTGGAGACTGGAGCACACTG		

### Data analysis

Data analysis was performed using GraphPad Prism v7.0. The proportion of fish positive for viable ISAv was analyzed using non-parametric Kruskal-Wallis analysis with Dunn’s multiple comparison test. Survival curves were analyzed using survival curve comparisons with Log-rank Martel-Cox test. The gene expression results were analyzed using BioRad CFX Manager v3.0 software. Primer efficiencies (E = 10^(-1/slope)^) were determined by analysis of 10-fold serial dilutions for the reference genes and 5-fold serial dilutions for the genes of interest using pooled cDNA. The mean efficiencies were 2.03±0.03 for all genes. The stability of EF-1Ab, RSP 20, 18 S and elF as reference genes was evaluated via the qBasePLUS GeNorm application [42]. All four reference genes were deemed to be stable, and therefore used to calculate the mean normalized relative quantity (MNRQ) of the target gene transcripts (M value <1.0, Coefficient of variation [CV] <0.5). Prior to statistical analysis, all samples were further normalized to basal samples taken prior to any infections (n = 24). Any “Tank Effect” within the experimental design was analyzed, prior to further statistical analysis, by a 2-way ANOVA. In all cases no statistical significance between groups present in each tank was seen and no “Tank Effect” could be observed.

## Results

### Sea lice infestation intensity

**Fig 2:**
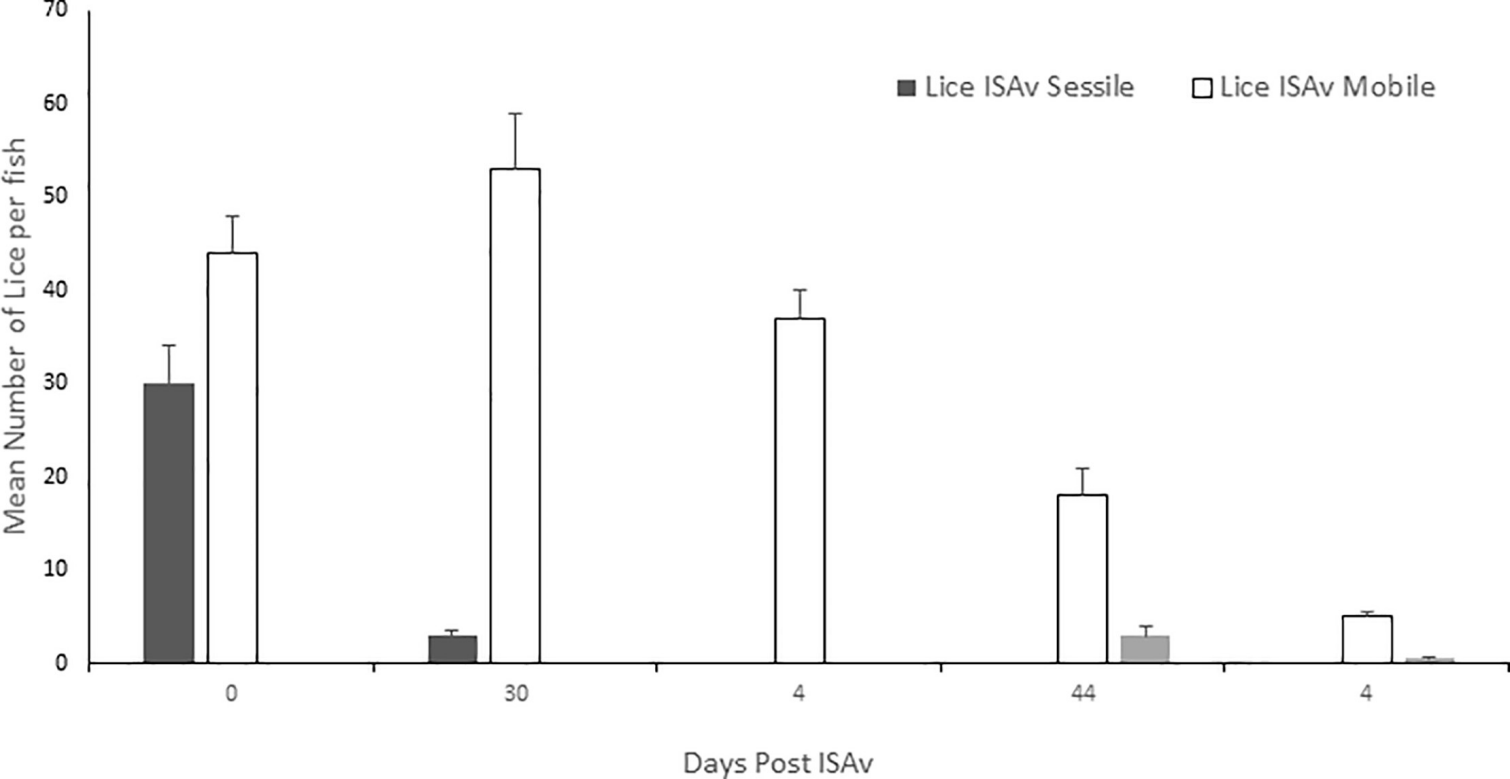
Mean number of sessile, mobile or gravid sea lice per Atlantic salmon pre- (day 0) and 3, 16, 37 and 51 days post—ISAv cohab infection which corresponds to 17, 21, 34, 55 and 69 days post-lice infection, respectively.

The ISAV cohabitation exposure began at 17 days post-lice infestation (d.p.l.i., Fig 2). This was chosen as the point at which to expose the fish to ISAv as lice cause the most host damage during the motile stages. Gravid female lice were only present on the fish at the later sampling time points of 37 and 51 days post-exposure (d.p.e.) to shedder fish (ISAv^+^ cohab), i.e. 55 and 69 d.p.l.i. (Fig 2). No lice were found on any fish sampled within the control or ISAv only treatment groups.

### Proportion of salmon positive for viable ISAv

**Fig 3:**
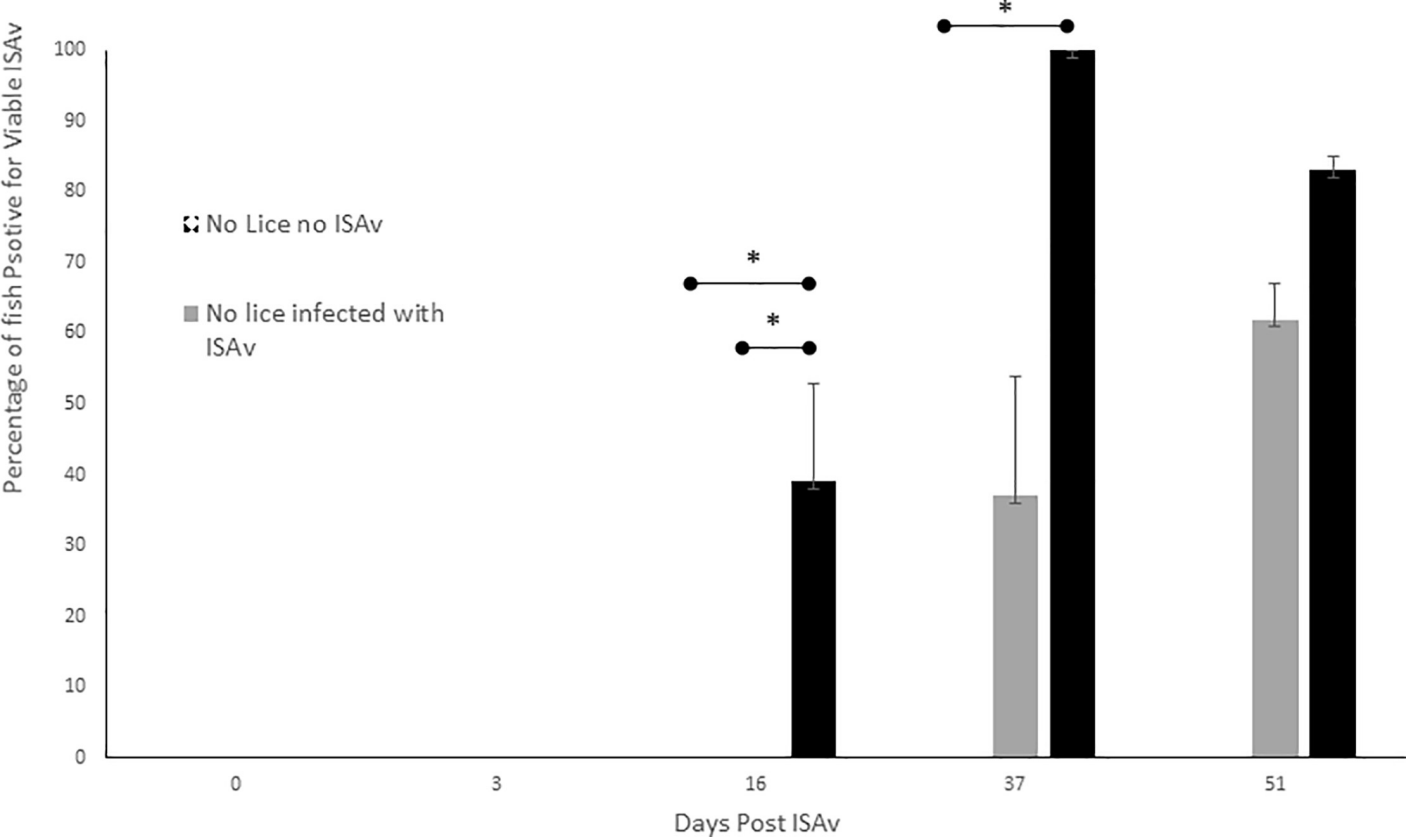
Mean percentages of Atlantic salmon positive for ISAv by cell culture pre- and 3, 16, 37 and 51 days post—ISAv cohab infection. Fish sampled at 51 d.p.e. to ISAv cohab fish were survivors of the ISAv infection. Data is combined from four replicate tanks per treatment with a total n per treatment group of 24 except in the lice plus ISAv treatment group at 37 d.p.e. to ISAv-infected cohab fish where n = 3 due to high mortalities and low survival in this treatment group. At 51 d.p.e. to ISAv-infected cohab fish i.e. survivor fish n = 45, 44 and 14 respectively.

Atlantic salmon pre-infested with *L*. *salmonis* were positive for viable ISAv earlier, at 16 d.p.e. to ISAV^+^ shedder fish than those without a prior sea lice infestation (ISAv only), which first appeared positive for viable ISAv at 37 d.p.e. to ISAv^+^ shedder fish (Fig 3). The proportion of salmon, with a prior sea lice infestation, positive for viable ISAv was only significantly higher than that of fish without a prior sea lice infestation at 16 days post-exposure to ISAv^+^ shedder fish (p < 0.05, Fig 3). The proportion of salmon with a prior sea lice infestation positive for viable ISAv was however, significantly higher than that of fish at 16 and 37 d.p.e to ISAV^+^ shedder fish (p < 0.05) even though there was no significant difference between that of the control group and the no lice plus ISAv group at either of these time points. Fish sampled 51 d.p.e. to ISAV^+^ shedder fish had survived ISAv exposure but were found to be carriers of viable ISAv. There was no significant difference between the proportion of ‘survivor’ fish positive for viable ISAv in the groups with or without a prior sea lice infestation (Fig 3).

ISAv presence was confirmed by both RT-PCR testing of the cell culture samples and qRT-PCR of head kidney samples from the corresponding fish for ISAv segment 8 RNA (snow eta al 2006). A sample was determined to be positive if the Ct value was <37 cycles, and therefore, Ct values >37 were considered negative for the presence of ISAv RNA.

### Percent survival of salmon

**Fig 4:**
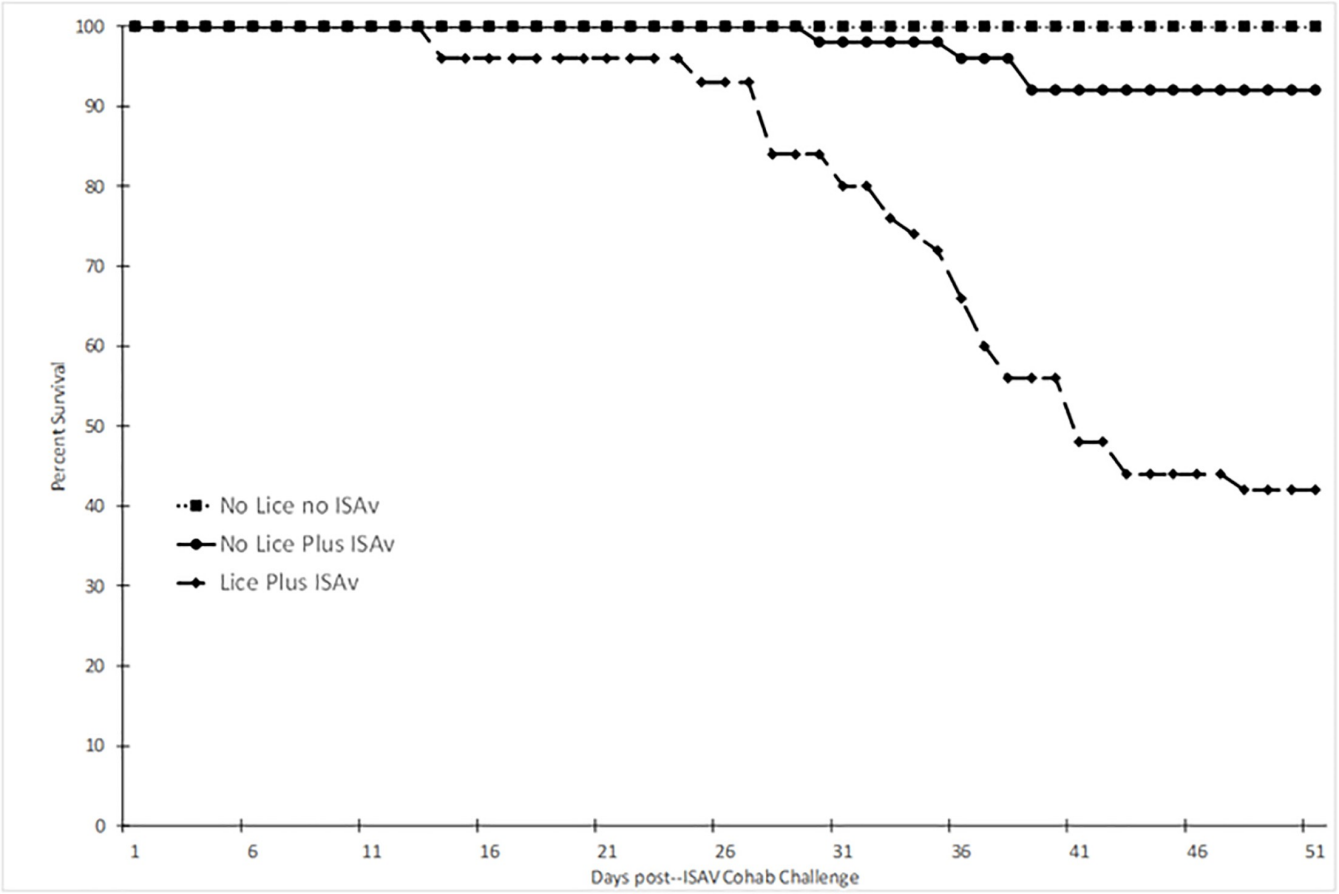
Percent survival of Atlantic salmon with and without a prior *L*. *salmonis* infestation post–exposure to ISAv-infected cohab fish (only mortalities which were positive for viable ISAv were included). Data combined from duplicate tanks per treatment group: no lice no ISAv, no lice plus ISAv and lice plus ISAv (n = 47, 48 and 33, respectively).

Atlantic salmon infested with *L*. *salmonis* prior to exposure to ISAV^+^ shedder fish possessed a significantly lower percent survival post-exposure to ISAV^+^ shedder fish than those without a prior sea lice infection (no lice plus ISAv group, p < 0.0001, Fig 4). Survival was also monitored for the control group of fish, which were sham-infested with sea lice and sham-exposed to ISAv (i.e. shedders i.p. injected with PBS, ISAV^-^). The survival of salmon without a prior sea lice infection post-exposure to ISAV^+^ shedder fish was also significantly lower than those not exposed to sea lice or ISAv^+^ shedder fish (p = 0.0443, Fig 4).

**Fig 5:**
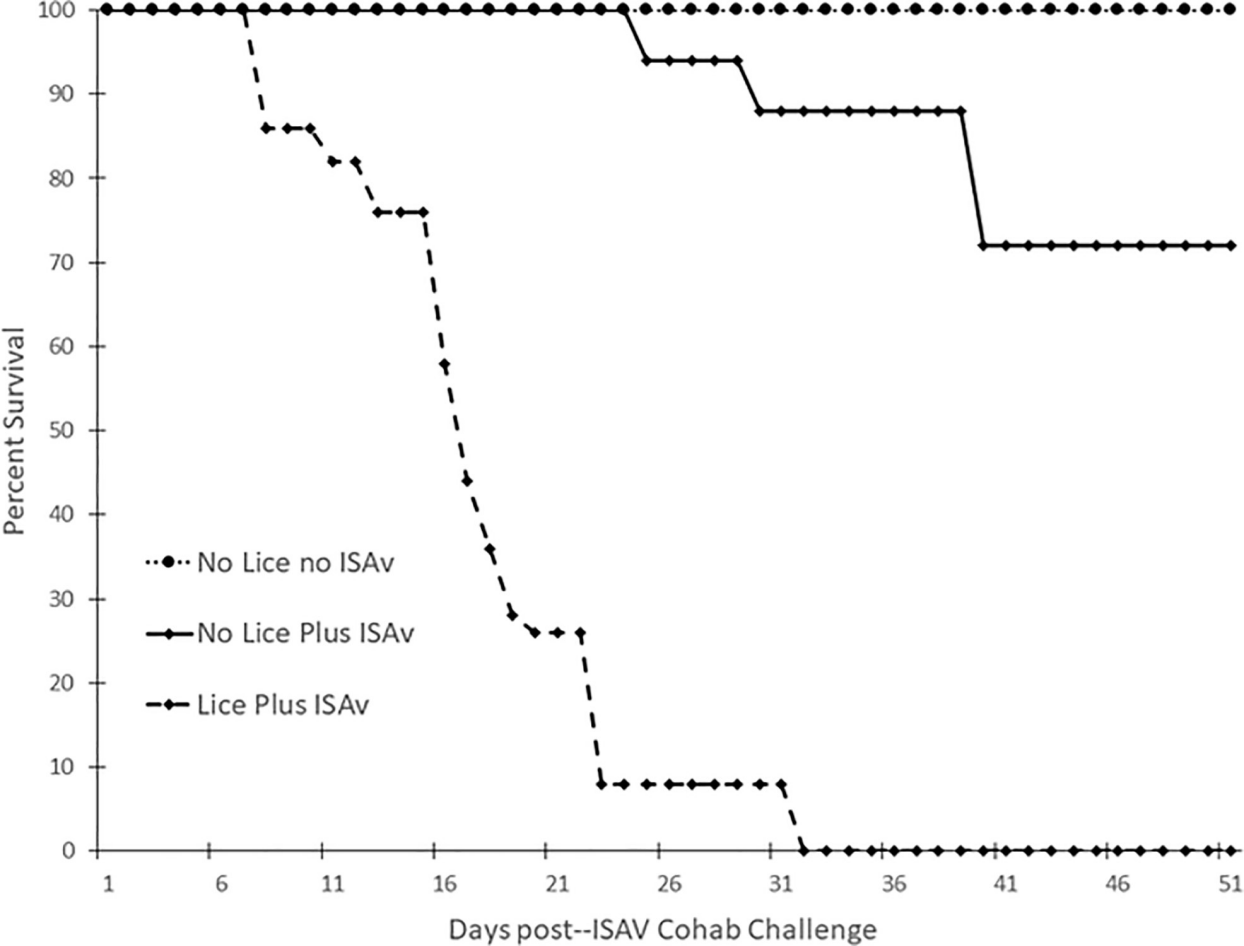
Percent survival of shedder Atlantic salmon with and without a prior *L*. *salmonis* infestation post—ISAv intra-peritoneal injection. The data is combined from six replicate tanks per treatment (n = 18 per treatment).

Shedder fish infested with *L*. *salmonis* prior to i.p. injection of ISAv (ISAv^+^) had a significantly lower percent survival than those without a prior sea lice infestation (p < 0.0001, Fig 5). Survival was also monitored for the ISAv^-^ shedder fish, which were sham-infested with sea lice and sham-exposed with ISAv. The percent survival of salmon with a prior sea lice infestation, post-i.p. injection with ISAv, was also significantly lower than those not exposed to sea lice or ISAv (p < 0.0001, Fig 5).

### Lice related gene expression

**Fig 6:**
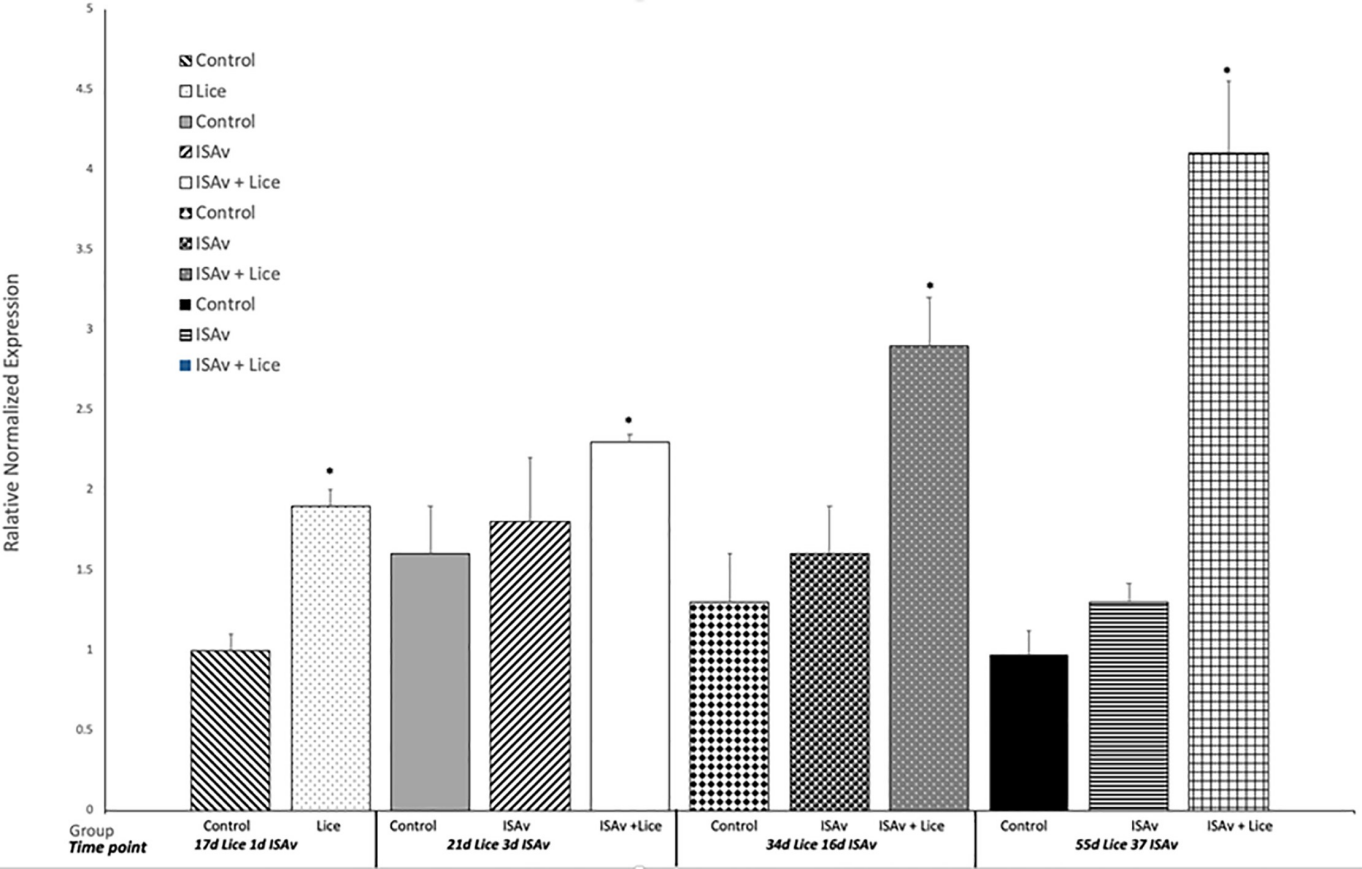
Relative normalized expression of the pro-inflammatory cytokine IL1-β in the anterior kidney of Atlantic salmon exposed to both sea lice and ISAv infections. The three treatment groups (control, ISAv only and ISAv + lice) were sampled at 4 separate time points (17 days post exposure (dpe) to sea lice and minus 1 dpe to ISAv, 21 dpe to lice and 3 dpe to ISAv, 34 dpe to lice and 16 dpe to ISAv and 55 dpe to lice and 37 dpe to ISAv). The asterisk indicates a significant difference between the control and treatment groups for that particular time point (p < 0.05, n = 24).

**Fig 7:**
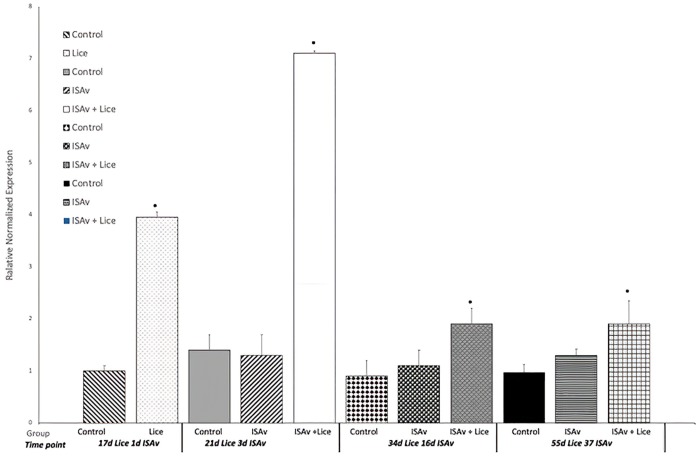
Relative normalized expression of the wound healing gene MMP 9 in the anterior kidney of Atlantic salmon exposed to both sea lice and ISAv infections. The three treatment groups (control, ISAv only and ISAv + lice) were sampled at 4 separate time points (17 days post exposure (dpe) to sea lice and minus 1 dpe to ISAv, 21 dpe to lice and 3 dpe to ISAv, 34 dpe to lice and 16 dpe to ISAv and 55 dpe to lice and 37 dpe to ISAv). The asterisk indicates a significant difference between the control and treatment groups for that particular time point (p < 0.05, n = 24).

**Fig 8:**
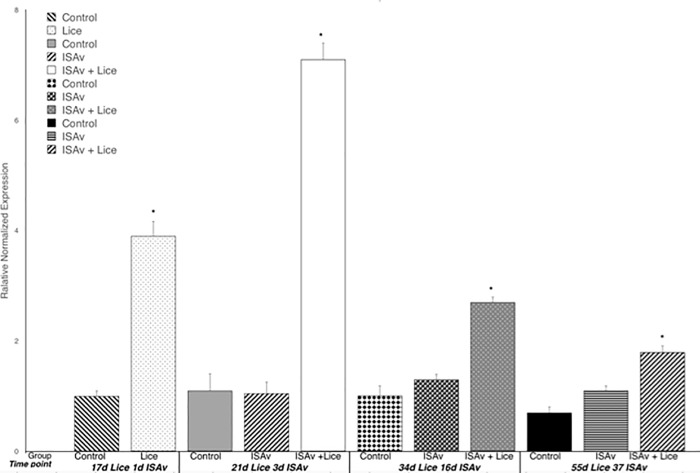
Relative normalized expression of the pro-inflammatory cytokine IL1-β in the anterior kidney of Atlantic salmon exposed to both sea lice and ISAv infections. The three treatment groups (control, ISAv only and ISAv + lice) were sampled at 4 separate time points (17 days post exposure (dpe) to sea lice and minus 1 dpe to ISAv, 21 dpe to lice and 3 dpe to ISAv, 34 dpe to lice and 16 dpe to ISAv and 55 dpe to lice and 37 dpe to ISAv). The asterisk indicates a significant difference between the control and treatment groups for that particular time point (p < 0.05, n = 24).

**Fig 9:**
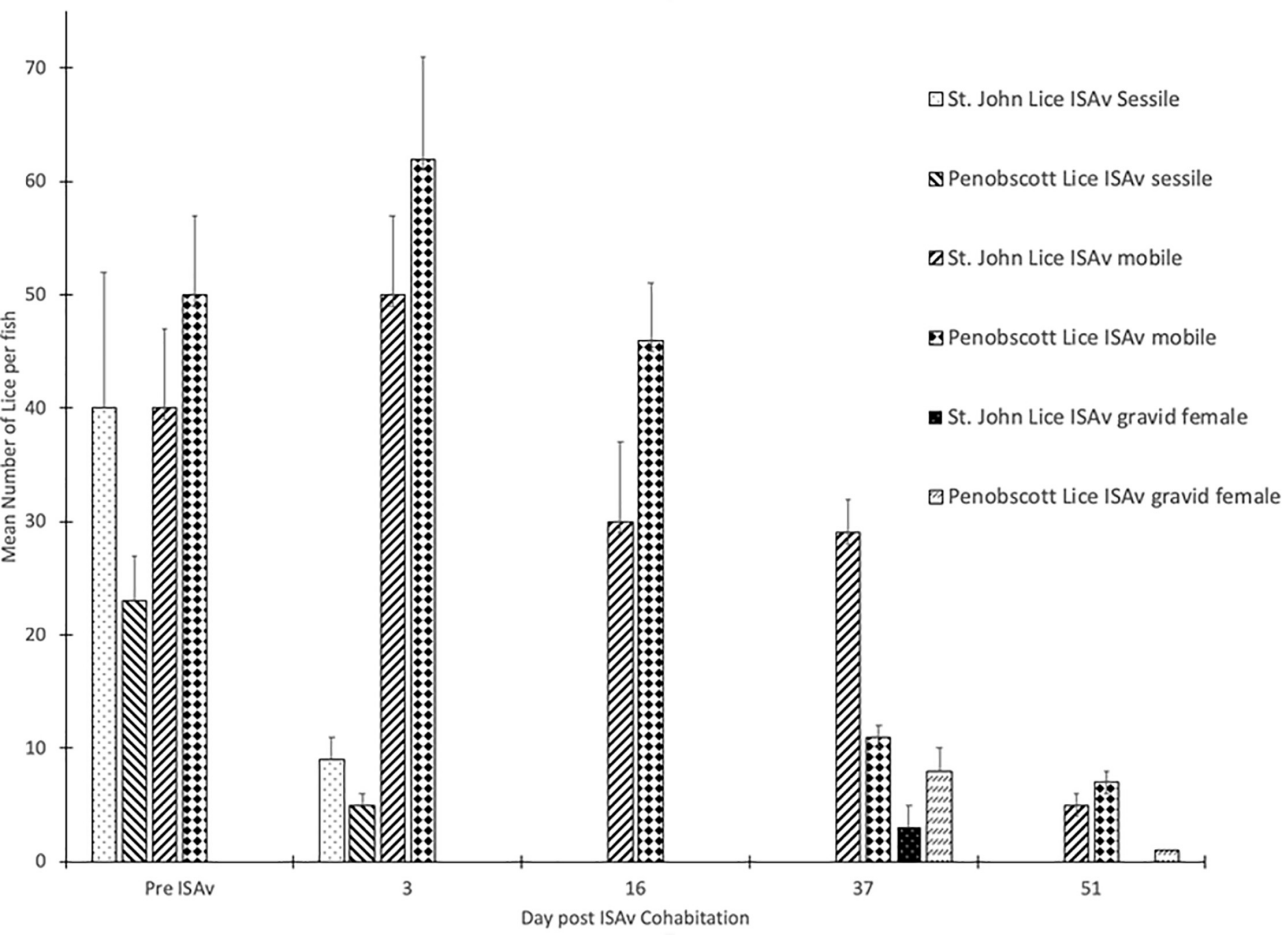
Mean number of sessile, mobile or gravid sea lice per Atlantic salmon from three combined families of St John or Penobscot derived stocks pre- and 3, 16, 37 and 51 days post—ISAv cohab infection which corresponds to 17, 21, 34, 55 and 69 days post-lice infection, respectively. No significant differences in sessile, mobile or gravid lice per fish between the two stocks at any of the time points sampled.

Gene expression results for IL-1β and MMP 9 were used to evaluate the response of the fish to sea lice infestation. It was found that the groups infested with sea lice had significantly higher levels of the pro-inflammatory cytokine IL-1β and MMP 9 across all time points than did the control and ISAv only groups (Figs 6 and 7). When the groups were broken down into their stock origins (Penobscot or Saint John), it was found that the combined three families of the Penobscot strain had significantly higher levels of IL-1β expression at 17 d.p.e to sea lice, i.e. prior to ISAv exposure, than did the three combined families of the Saint John strain (Fig 8). However, there was no significant differences between the lice counts per fish on the Saint John versus the Penobscot derived families regardless of louse life stage (Fig 9). The expression of IL-1β in the Saint John strain families was however, significantly higher than the Penobscot strain families at 21 dpe to lice and 3 d.p.e. to ISAv (Fig 8).

### ISAv related gene expression

**Fig 10:**
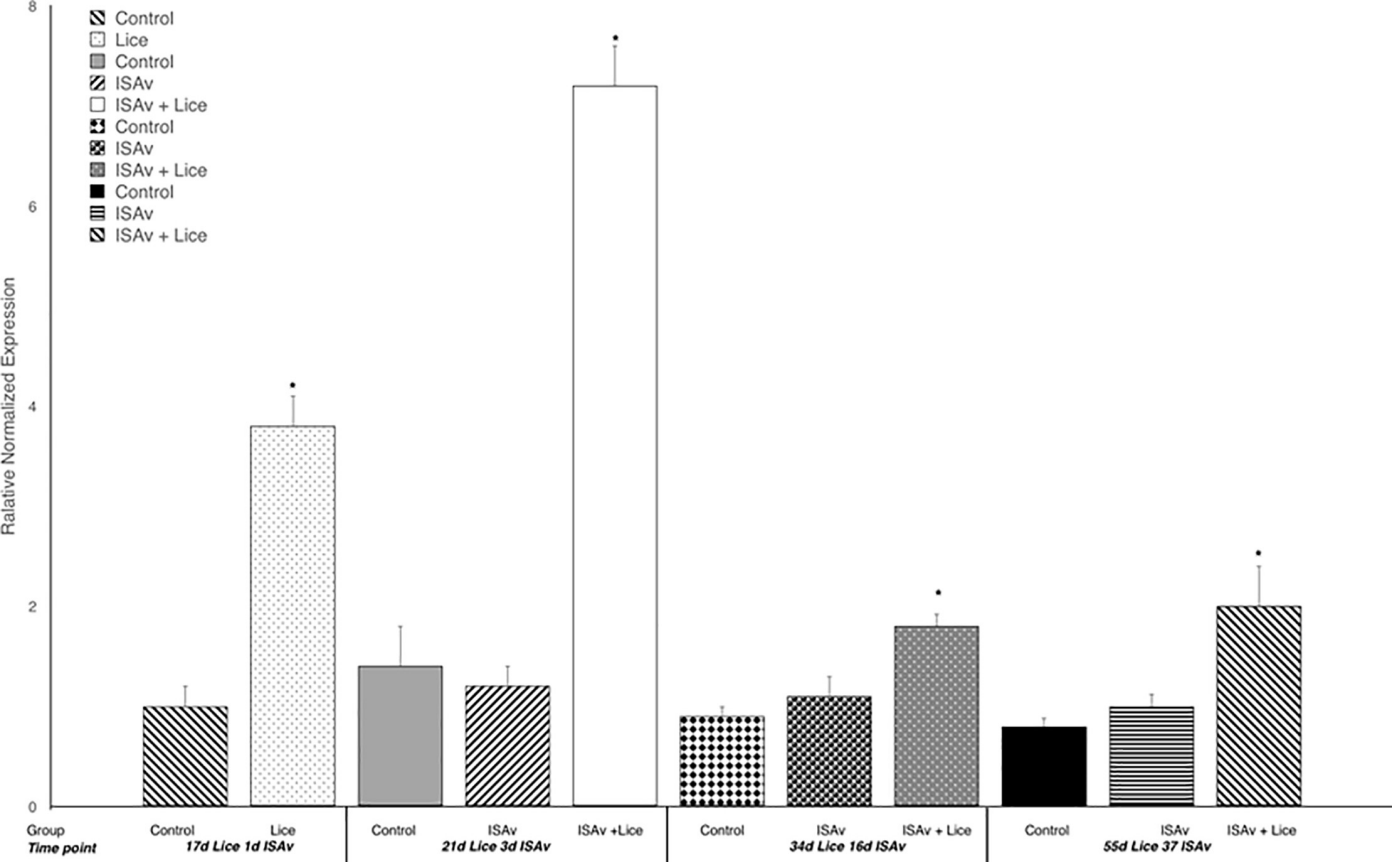
Relative normalized expression of Mx-1 in the anterior kidney of Atlantic salmon exposed to both sea lice and ISAv infections. The three treatment groups (control, ISAv only and ISAv + lice) were sampled at 4 separate time points (17 days post exposure (dpe) to sea lice and minus 1 dpe to ISAv, 21 dpe to lice and 3 dpe to ISAv, 34 dpe to lice and 16 dpe to ISAv and 55 dpe to lice and 37 dpe to ISAv). The asterisk indicates a significant difference between the control and treatment groups for that particular time point (p < 0.05, n = 24).

**Fig 11:**
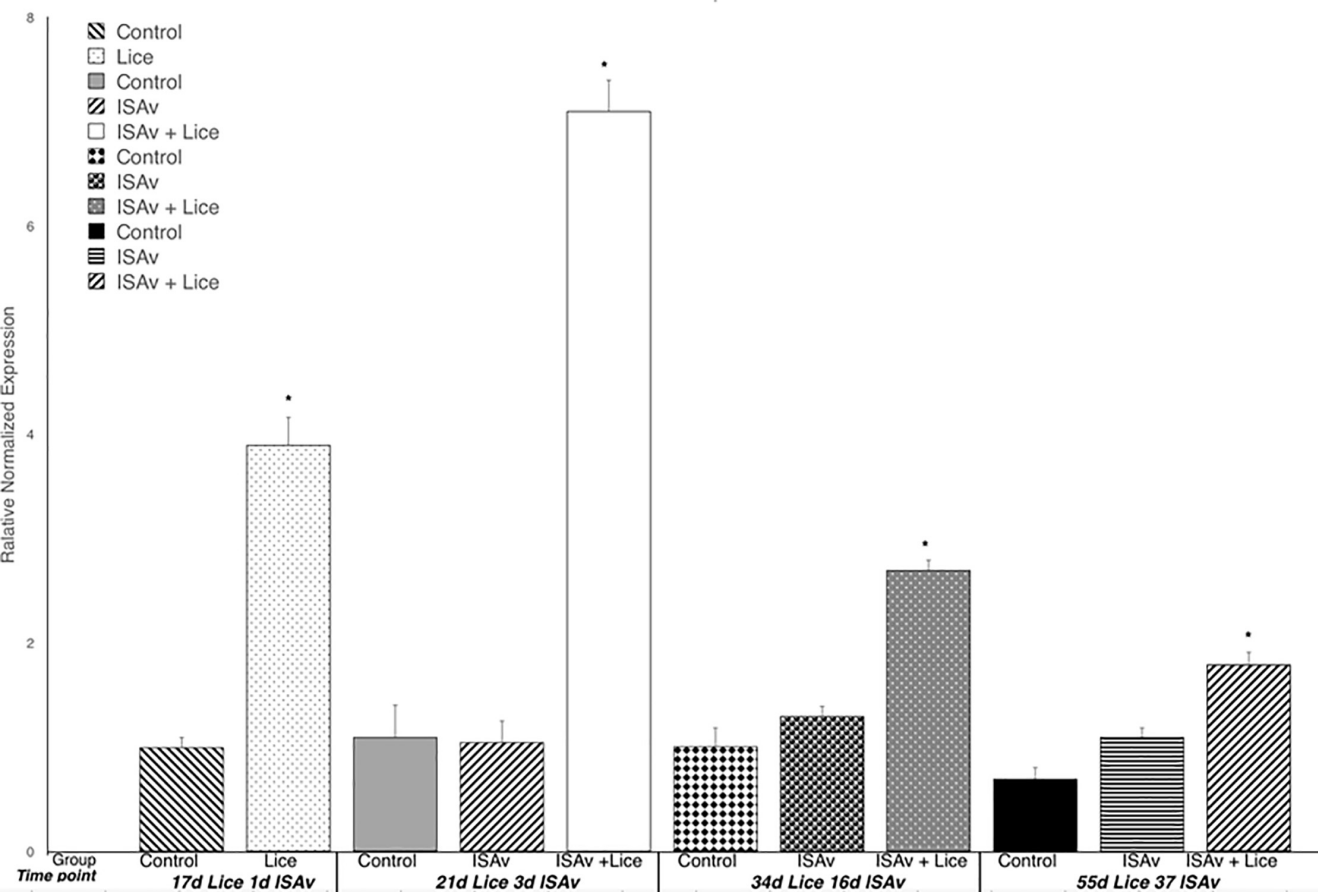
Relative normalized expression of Mx-1 in the anterior kidney of Atlantic salmon exposed to both sea lice and ISAv infections. **The three treatment groups (control, ISAv only and ISAv + lice) were sampled at 4 separate time points (17 days post exposure (dpe) to sea lice and minus 1 dpe to ISAv, 21 dpe to lice and 3 dpe to ISAv, 34 dpe to lice and 16 dpe to ISAv and 55 dpe to lice and 37 dpe to ISAv).** The asterisk indicates a significant difference between the control and treatment groups for that particular time point (p < 0.05, n = 24).

**Fig 12:**
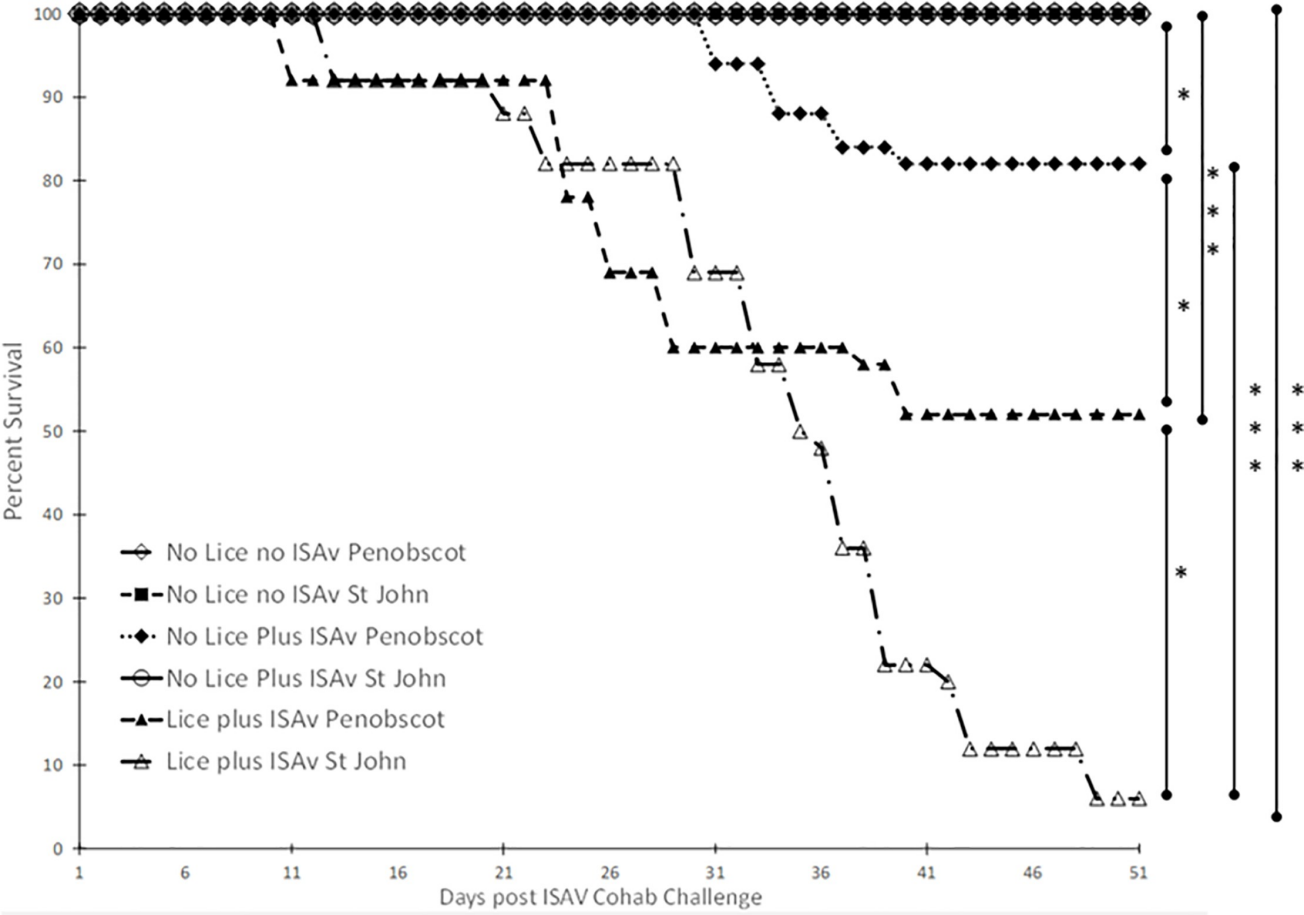
Percent survival of St John- and Penobscot- derived Atlantic salmon with and without a prior *L*. *salmonis* infection post–exposure to ISAv-infected cohab fish. Data combined from duplicate tanks per treatment group: no lice no ISAv, no lice plus ISAv and lice plus ISAv (St John versus Penobscot derived fish, n = 15 and 29, n = 23 and 25, n = 17 and 26, respectively).

At 37 dpe to ISAv-infected cohab fish, the group with both lice and ISAv had a significantly higher expression of Mx1 than did the ISAv only and control groups (Fig 10). When the treatment groups were broken down into the two stock origins, it was found that there was a significantly higher level of Mx1 expression in the three combined families of St John derived fish exposed to ISAv at 3 dpe than that observed in the three combined families of the Penobscot strain tested (Fig 11). It was also found that the three combined Saint John families tested possessed significantly lower survival post-ISAv exposure than the Penobscot strain whether a prior lice infection was present or not (p = 0.0211 and p = 0.0309, respectively, Fig 12). There was still a significantly lower survival observed in both the St John and the Penobscot stocks which were exposed to *L*. *salmonis* prior to ISAv exposure in comparison to fish only exposed to ISAv (p < 0.0001 for both, Fig 12).

**Fig 13:**
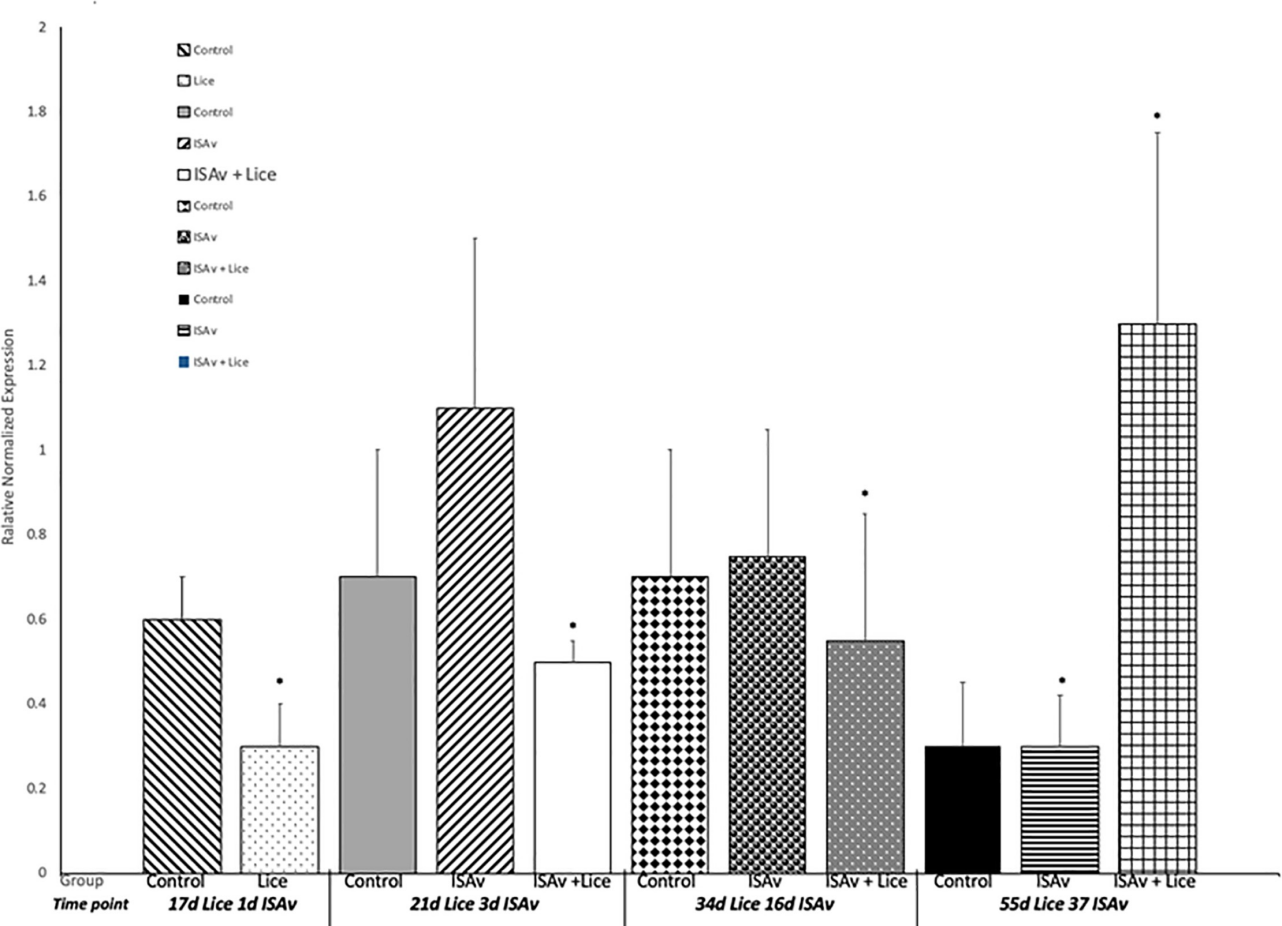
Relative normalized expression of MHC class I β in the anterior kidney of Atlantic salmon exposed to both sea lice and ISAv infections. The three treatment groups (control, ISAv only and ISAv + lice) were sampled at 4 separate time points (17 days post exposure (dpe) to sea lice and minus 1 dpe to ISAv, 21 dpe to lice and 3 dpe to ISAv, 34 dpe to lice and 16 dpe to ISAv and 55 dpe to lice and 37 dpe to ISAv). The asterisk indicates a significant difference between the control and treatment groups for that particular time point (p < 0.05, n = 24).

At time points minus 1, 3, and 16 d.p.e. to ISAv ^+^ shedders, the groups infested with lice had significantly lower levels of MCH1-ß expression than did both the control and ISAv only groups (Fig 13). An increase in MHC1-ß expression did not occur until 37 dpe to ISAv in the lice + ISAv infected group.

**Fig 14:**
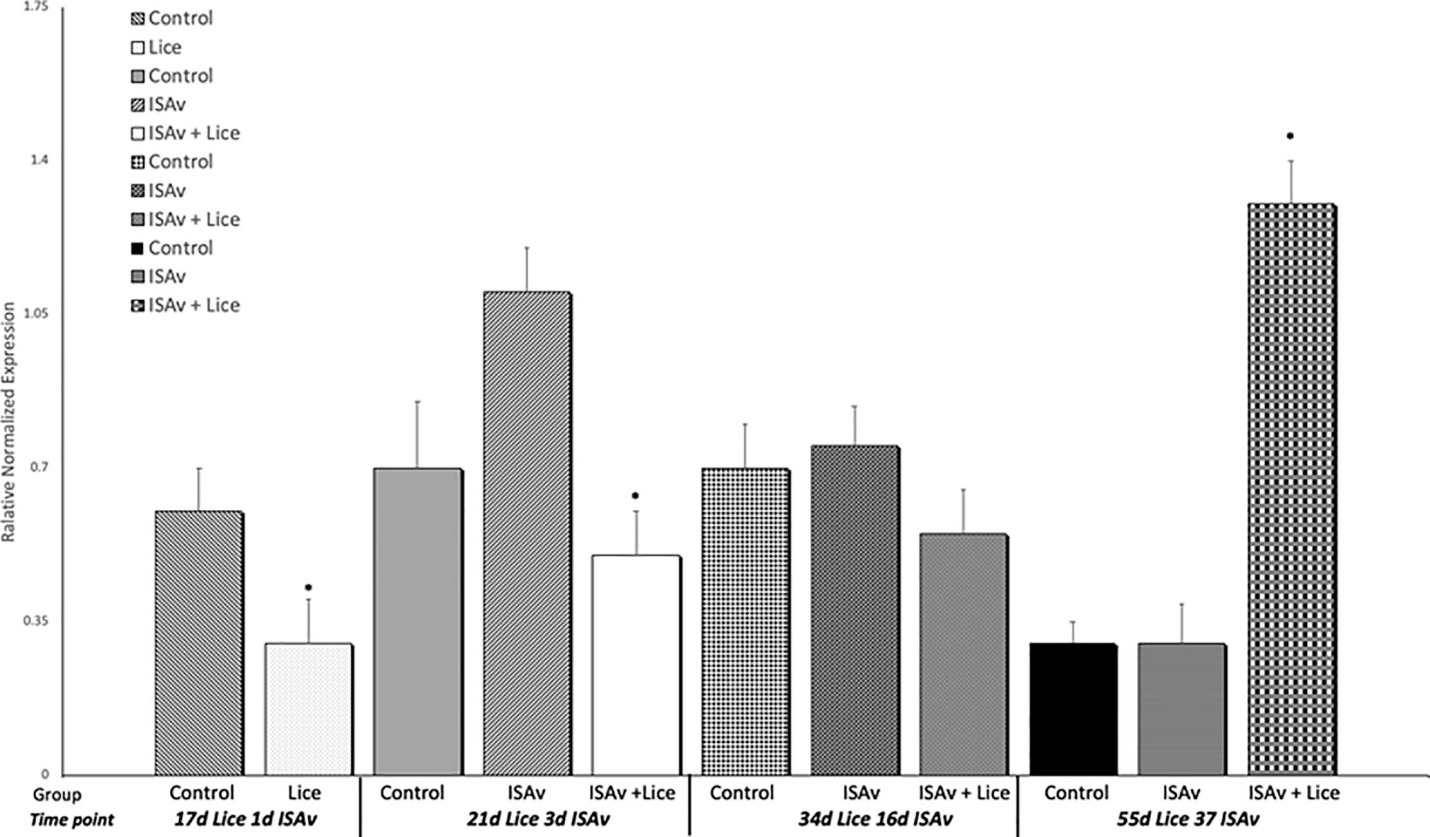
Relative normalized expression of ISAv responsive gene galectin 9 in the anterior kidney of Atlantic salmon exposed to both sea lice and ISAv infections. The three treatment groups (control, ISAv only and ISAv + lice) were sampled at 4 separate time points (17 days post exposure (dpe) to sea lice and minus 1 dpe to ISAv, 21 dpe to lice and 3 dpe to ISAv, 34 dpe to lice and 16 dpe to ISAv and 55 dpe to lice and 37 dpe to ISAv). The asterisk indicates a significant difference between the control and treatment groups for that particular time point (p < 0.05, n = 24).

The gene expression levels of galectin 9, TRIM 16 and TRIM 25 were all used as a measure of an appropriate response by the fish host to ISAv infection. The pattern of expression for all 3 genes, Galectin-9, TRIM 16 and TRIM 25, was similar across groups and time points. At both minus 1 and 3 dpe to ISAv, the lice infested groups showed a down-regulation of Galectin 9 gene expression in comparison to the control and ISAv only groups (Fig 14). By 16 dpe to ISAv, the lice plus ISAv infected group had its level of Galectin 9 return to levels similar to both the control and ISAv only groups (Fig 14). Again, similar to other genes measured, galectin-9 was significantly higher in the lice + ISAv group than all other fish on the final sampling day (Fig 14). The same pattern held true for TRIM 16 and TRIM 25 gene expression except that TRIM 25 gene expression of the lice infested group continued to show lower gene expression at 16 dpe to ISAv in comparison to both the control and ISAv only group.

There were no significant differences in Galectin-9, TRIM 16 or TRIM-25 gene expression between the Penobscot and the Saint John derived stocks of salmon examined at any of the sample points within a certain treatment group.

## Discussion

This is the first laboratory study examining impacts of *Lepeophtheirus salmonis* infestation on subsequent susceptibility of salmon to ISAv. While infestation with sea lice has long been implicated as a vector for ISAv and/or compromising host ability to respond to co-infection with viral challenge [43–45], this is the first study to show conclusively that prior infection with sea lice increases the susceptibility of salmon to ISAv. The proportion of salmon with a prior sea lice infestation that were positive for viable ISAv was significantly higher than that of control no lice no ISAv fish at 16 and 37 d.p.e to ISAv cohab fish (p < 0.05) even though there was no significant difference between that of the control group and the no-lice plus ISAv group at either of these time points. This suggests that Atlantic salmon with a high load of motile *L*. *salmonis* were much more susceptible to ISAv infection via cohabitation as viable virus was detected at an earlier time point post-ISAv exposure. There was also no significant difference in survival between the control no-lice no-ISAV shedder group and the no-lice plus ISAV shedder group (p = 0.0713, Fig 5). This data suggests that Atlantic salmon possessing a high load of motile *L*. *salmonis* infestation are more susceptible to ISAv infection via i.p. injection due to the fact that these fish possessed higher mortality with no survival in comparison to the fish i.p. injected with ISAv without a prior lice infestation which had significantly lower mortality. The fact that the lice plus ISAv fish died faster with 100% mortality by 32 d.p. i.p. challenge may have resulted in virus being shed earlier or at a higher abundance in the cohabitation challenge tanks and hence the earlier detection of viable ISAv in the lice infested cohabitee fish at 16 d.p.e. to ISAv+ cohab fish.

### Virus isolation

Virus isolation using cell culture has been found to have a high specificity for ISAv but can have less sensitivity than qPCR McClure [44]. However, qPCR does not differentiate between viable and non-viable ISAv particles and can result in false positives reducing specificity. The qPCR assay employed in this study has been found to have a specificity of 99% [44]. Furthermore, McClure et al [44] suggested that when large sample sizes are screened, as in the current study, larger numbers of false positives may be generated. In the current study, all non-ISAv exposed fish tested negative for the presence of ISAv RNA, except for a single fish at 37 d.p.e and two mortalities, all of which occurred in the ISAv sham infection group. These three-fish tested positive for ISAv RNA by qRT-PCR but negative for viable virus, by cell culture and subsequent RT-PCR. No other mortalities occurred in the control group and the two positive by qPCR showed no clinical signs of ISAv. A possible explanation for the presence of low quantities of ISAv RNA in three fish from the control groups not co-habitated with ISAv injected fish is that they were exposed to non-viable ISAv that was shed by infected fish in the other tanks on the same recirculation system that was inactivated by UV sterilization process prior to entering the non-ISAv exposed tanks. These observations combined with zero other mortality in controls and high mortality levels in ISAv exposed groups, would suggest potential contamination and false positives. Overall, samples that were positive by qPCR for ISAv RNA, but not from a treatment group exposed to ISAv occurred at a rate of 1.3% which is in line with the 99% specificity as defined by Murray et al [46] and Snow [47]. It should be noted that no ISAv control groups tested positive for viable ISAv by tissue culture or showed clinical signs of ISA.

### Lice settlement and related gene expression

A high level of lice infestation was achieved in this study, starting with approximately 60–80 sessile chalimus at pre-ISAv sampling and achieving up to 50 mobile lice following the addition of shedder fish. However, the level of lice infestation dropped drastically from 16 days post-exposure to ISAv+ shedder fish. Lice and ISAv co-infection induced inflammatory and tissue remodeling genes IL-1 and MMP 9 throughout the study. Penobscot strain salmon had significantly higher levels of IL-1β expression at 17 d.p.e to sea lice, i.e. prior to ISAv exposure, than did the three combined families of the Saint John River, whereas the reverse was true at 21 dpe to sea lice, suggesting the three combined families of Penobscot strain tested were able to mount an inflammatory response to the sea lice more rapidly than the three combined families of the Saint John strain.

The same pattern of expression held true when investigating MMP 9 gene expression which was used as an indicator to evaluate the wound healing response of the fish to sea lice infestation Development, application and validation of a Taqman (R) real-time RT-PCR assay for the detection of infectious salmon anaemia virus [48, 49]. Again, the Penobscot strain had significantly higher levels of MMP 9 expression, this time at 34 d.p.e to sea lice than did the three combined families of the Saint John strain tested. The delay in upregulation of MMP9 in relation to IL-1 is expected as tissue re-modeling and wound repair are downstream in the initiation of an inflammatory response and have been observed under other lice infections in salmon [48, 49]. This did not appear to result in a lower lice load on the fish of these families. Whether this had any influence on the higher survival of these fish from the combined Penobscot derived families tested in comparison to the Saint John families tested post-ISAv exposure cannot be completely untangled from the greater inherent resistance in the Penobscot strain compared to Saint John river strain against ISAv, since the Penobscot strain had higher survival than the Saint John River strain under both conditions and exhibited parallel increases in susceptibility to ISAv based on lice exposure. This observation may be explained because fish that are upregulating inflammation and wound healing may heal wounds caused by the lice more rapidly. The more rapidly healing wounds prevent the ISAv penetrating the host as easily when compared to fish with open wounds in the epidermis. Such a model for MMP 9 has been suggested by several groups [22, 48–50] as a possible mechanism for sea lice resistance in Atlantic salmon [51, 52].

### ISAv related gene expression

Mx is an interferon induced antiviral protein and ISAv has been found to induce a strong Mx response both in vitro and in vivo [53, 54]. However, even though key type I interferon induced proteins such as Mx are induced they are not considered protective and unable to inhibit ISAv replication either in vitro or in vivo [53, 54]. Gene expression of Mx1 indicates exposure to ISAv and is commonly upregulated in vertebrates to orthomyxoviral exposure (part of the ‘influenza pathway’ [55, 56], but is not indicative of an appropriate ISAv immune response [53, 54, 57, 58], and can also be observed to be impacted under other viral or intracellular bacterial infections [59–61]. In this study, it was found that Mx1 expression was significantly down-regulated in the lice exposed group at 17 dpe (i.e. just prior to ISAv exposure) as compared to the control group. The findings may indicate that exposure to sea lice infection prior to viral infection hampers the ability of the fish to recognize subsequent ISAv/intracellular pathogen exposure.

At 37 dpe to ISAv-infected cohabitation fish, the group with both lice and ISAv had a significantly higher expression of Mx1 than did the ISAv only and control groups, suggesting a delayed immune response to ISAv exposure in the dual infected fish as energy was also being utilized to mount an anti-parasitic response to the lice infestation. In the lice plus ISAv group, at 37 d.p.e. to ISAv, only three fish remained at this time point, due to the high ISAv specific mortality in the dual infection groups. Hence, any conclusions drawn at 37 d.p.e. to ISAv in this treatment group must be made with caution.

When the treatment groups were broken down into the two stock origins (Penobscot and Saint John), it was found that there was a significantly higher level of Mx1 expression in the three combined families of Saint John derived fish exposed to ISAv at 3 dpe than that observed in the three combined families of the Penobscot strain tested. This may suggest that these combined families of Saint John origin are more capable of early recognition of ISAv than the combined families of Penobscot origin examined. However, it must be remembered that Mx1 up-regulation is not protective [58, 62]and does not inhibit ISAv replication [58, 62]. It was also found that the three combined Saint John families tested possessed significantly lower survival post-ISAv exposure whether a prior lice infestation was present or not (p = 0.0211 and p = 0.0309, respectively). This data suggests that the three combined families of Saint John stock tested were more susceptible to mortality due to ISAv and that the up-regulation of Mx1 observed in these fish at 3 dpe to ISAv was not protective as indicated for Mx1 upregulation in previous studies [53, 54]. There was still a significantly lower survival rate observed in both the Saint John and the Penobscot stocks which were exposed to *L*. *salmonis* prior to ISAv exposure in comparison to fish only exposed to ISAv (p < 0.0001 for both).

For instance, pro-inflammatory genes like IL-1are known to be increased due to ISAv infection and in some cases down regulated in salmon infections. Other genes such as MH class I, Galectin, TRIM, Mx, genes are all responsive to viral infection. As IL-1 can be differentially regulated by ISAv or Lsal, under co-infection, we might expect either direction of expression. Furthermore, as the fish become moribund and may be close to dying, gene disregulatin is common, and responses may not be functional at this time.

MHC1-β expression was significantly down-regulated in the groups infested with lice at days -1, 3 and 16 dpe to ISAv. There was no increase in MHC1-β expression in the lice infested group until 55 dpe to lice and 37 dpe to ISAv. This may suggest that prior lice infestation impairs the ability of the fish to mount a MHC1-β response to ISAv exposure. MHC1 is involved in intracellular antigen presentation, e.g. viral peptides, at the surface of infected cells. Any down-regulation in MHC1-ß expression early in the infection may reduce the efficiency of antigen presentation and T cell activation. Jorgensen et al. [63] suggest that CD8+ T cells, which recognize viral targets by binding to viral antigens associated with MHC class I on infected cells, are important during early ISAv viraemia, in comparison to CD4 + T cells. Genes chosen for analysis were based on the literature on sea lice and ISAv. The down-regulation in MHC1-ß gene expression corresponds with a down-regulation of MHC1-ß protein expression. This process may also impact antigen presentation in ISAv and sea lice infected fish, but this will require further testing to confirm. There were no significant differences in MHC1- ß expression levels between the Penobscot and St John stocks across any of the time points sampled.

The gene expression levels of Galectin 9, TRIM 16 and TRIM 25 were used as a measure of the host anti-viral response. Galectin-9 is a non-membrane bound soluble lectin which is up-regulated during ISAv infection of ASK cells in vitro [64] and is thought to play a role in the regulation of a diverse array of immune functions [65]. Schiotz et al. [66] have also observed the up-regulation of galectin-like genes post-ISAv infection in vitro and stated that it is a marked feature of ISAv infection in vitro. Tripartite motif-containing protein (TRIM) family members such as TRIM 16 and TRIM 25 are known to be up-regulated 3, 7 and 16 days post-ISAv infection in salmon [62], and although their exact functions in fish are unknown they are hypothesized to play a role in modulating fundamental innate anti-viral immune responses. For instance, Gack et al [67] and LeBlanc [68] et al suggested that TRIM 25 is involved in IFN-ß production, and another TRIM called TRIM 5α has been found to interfere with HIV-1 replication in primates [69]. The pattern of expression for all 3 genes, Galectin-9, TRIM 16 and TRIM 25, was similar across groups and time points. At both pre-exposure and 3 dpe to ISAv, the lice infested groups showed a down-regulation of anti-viral gene expression in comparison to the control and ISAv only groups. By 16 dpe to ISAv, the lice plus ISAv infected groups level of Galectin 9 had returned to levels similar to the controls and ISAv only groups. This pattern was also seen for TRIM 16 gene expression. These results suggest that prior infestation with sea lice impairs the ability of the fish to mount an appropriate early anti-viral response to subsequent ISAv exposure. The only difference with regard to the pattern of TRIM 25 gene expression was that the lice infested group continued to show lower gene expression at 16 dpe to ISAv in comparison to both the control and ISAv only group.

### Relationship to ISAv infections in farmed populations of Atlantic salmon

Sea lice infestation typically occurs shortly after introduction to the marine sites in Eastern Canada and the United States. Whereas ISAv outbreaks often take much longer to occur. For example, the Hammell and Dohoo [70,71] (2005ab) papers showed that risk of mortalities due to ISAv were increased due to presence of sea lice and McClure et al (2005) [72] also showed that significant risk factors for ISAv outbreaks involved the number of times that the salmon were treated for sea lice. Finally, ISAv presence in a farm often requires depopulation of the infected cage, and therefore investigation of lice infestations following ISAv exposure, did not appear to make any sense in terms of application to a field setting. In terms of infestation levels, the reviewers are guided to data from the Atlantic salmon fish farmers Assocaition (ACFFA) reports for Bay of Fundy (BoF) Canada, from 2010 to present. Infestation levels in the BoF often reach 5–10 adult females per fish and in some cases > 20 adult females per fish. Data for adult males, pre-adults and chalimus stages in these reports are not given, but gender ratios are always close to 50:50 and numbers of other stages generally are higher than for adult females, aside from over wintering seasons. We chose the infestation level we did to be in the same range as BoF, Canada.

## Conclusions

Salmon pre-infested with a high intensity of late chalimus and pre-adult *L*. *salmonis* prior to ISAv exposure appear more susceptible to ISAv infection via cohabitation infection, which mimics a natural route of infection, as well as via i.p. injection. Viable ISAv was detected earlier in cohabitee fish with a prior lice infestation at 16 d.p.e. to ISAv compared to 37 d.p.e. to ISAv in the cohabitee fish without sea lice. Higher mortality with no survival was observed in the dual infection shedder fish which received an i.p. injection of ISAv in comparison to the fish i.p. injected with ISAv without a prior lice infestation which had significantly lower mortality. The earlier inflammatory and wound healing signals (i.e. IL-1 and MMP 9) and lack of Mx responses in Penobscot strain fish were associated with higher survival to co-infection compared to Saint John strain families, however, Penobscot strain families also exhibited inherently greater resistance to ISAv compared to Saint John strain families regardless of single ISAv or co-infection with sea lice. In all cases, prior sea lice infestation reduced systemic anti-viral responses, and likely contributed to the faster uptake of ISAv and higher mortality levels of infected salmon. Further research into the relative contribution of protective mechanisms against ISAv in Penobscot strain families is warranted, as is the need to determine whether the level of sea lice infestation impacts the degree of enhanced susceptibility to ISAv or other viral infections in salmon.

## Supporting information

S1 File Figure and Table Summary Files. (XLSX)

S2 File Gene Expression. Gene expression SEM summary data.
(XLSX)
