# Correction: Sea lice, *Lepeophtheirus salmonis* (Krøyer 1837), infected Atlantic salmon (*Salmo salar* L.) are more susceptible to infectious salmon anemia virus

**DOI:** 10.1371/journal.pone.0213232

**Published:** 2019-03-05

**Authors:** The PLOS ONE Staff

The third and fourth authors’ names are spelled incorrectly. The correct names are: Jennifer Covello, and Sara L. Purcell. The publisher apologizes for the errors. The correct citation is: Barker SE, Bricknell IR, Covello J, Purcell SL, Fast MD, Wolters W, et al. (2019) Sea lice, *Lepeophtheirus salmonis* (Krøyer 1837), infected Atlantic salmon (*Salmo salar* L.) are more susceptible to infectious salmon anemia virus. PLoS ONE 14(1): e0209178. https://doi.org/10.1371/journal.pone.0209178
